# Polyphenols in Agricultural Grassland Crops and Their Health-Promoting Activities—A Review

**DOI:** 10.3390/foods12224122

**Published:** 2023-11-14

**Authors:** Emily P. Verhulst, Nigel P. Brunton, Dilip K. Rai

**Affiliations:** 1 Department of Food BioSciences, Teagasc Food Research Centre Ashtown, D15 KN3K Dublin, Ireland; emily.verhulst@teagasc.ie; 2 The School of Agriculture and Food Science, University College Dublin, Belfield, D04 V1W8 Dublin, Ireland; nigel.brunton@ucd.ie

**Keywords:** grassland, agriculture, polyphenols, phenolic acids, flavonoids, antioxidant, anticancer, anti-diabetic

## Abstract

Grassland crops are emerging reservoirs of undisturbed, natural antioxidants and phytochemicals, such as phenolic acids and flavonoids. The present review will focus on the most commonly cultivated crops, namely *Lolium perenne* L, *Cichorium intybus* L, *Plantago lanceolata* L. and *Trifolium pratense* L, which have been recognized for their polyphenolic composition. However, these crops are often undervalued and underutilized, yet have the means of potentially creating novel, value-added food and nutraceutical products. Previous studies relating to these crops have identified them as rich sources of caffeic acid, chlorogenic acid, daidzein, kaempferol, luteolin, and quercetin. The key to harnessing the hidden potential of these species is the recovery, identification, and characterization of the phytochemicals they contain. Considering the upsurge of research studies on alternative plant-based diets for the health of humans and the planet earth, there is a necessity to understand the phytochemical composition and the bioactivity that they possess. This review summarizes recovery methods of phytochemicals from the aforementioned grassland crops and their compositional and functional (antioxidant, anti-cancer, and anti-diabetic) characterization and discusses the potential for grassland crops as an abundant reservoir of health-promoting ingredients which can increase the nutritional composition within novel food innovations or within nutraceuticals.

## 1. Introduction

Grassland crops are typically produced for the purpose of rearing agricultural livestock which is often comprised of multispecies grassland swards, including perennial crops, such as *Lolium*, *Cichorium*, *Plantago*, and *Trifolium* species, amongst others [1]. However, given the large volumes of biomass that grassland represent and global shortages in protein production, in addition to their role as forage, grassland may have the potential to contribute towards food security as an alternative source of proteins [2]. Proteins are typically recovered from plants via a series of steps involving the extraction of the protein, isolation via precipitation, and finally recovery by drying. This process results in the accumulation of residual, non-protein biomass, which is at odds with a circular approach to the sustainable utilization of natural resources. Given that residual biomass is likely to contain many components, which could be used for other functions in foods such as dietary fibers, polyphenols, lignins, pigments, and organic bio-compounds, it is of considerable research interest to examine these resources as a source of these compounds.

Plant-based diets provide essential nutrients required for life, and supply non-nutrient components that promote health, well-being, and reduce the risk of degenerative diseases [3]. It is for this reason that the majority of public health guidelines recommend the inclusion of at least 400g or five portions of fruit and vegetables per day. Some of the associated health-benefits arising from a diet of this nature could be associated with the presence of primary and secondary metabolites present in plants. While primary metabolites (proteins, carbohydrates, and vitamins) are essential components for a healthy diet, secondary metabolites are not considered essential. Within the plant, these metabolites have a ‘survival’ role, i.e., they act as a defense mechanism in response to environmental stress, or when competing with other plants, for example some plants exhibit bright colors or odors to promote pollination [4,5]. Over time plants have evolved a vast array of these defense chemicals and up to 100,000 different secondary metabolites can be found in plants belonging to a number of different classes, including polyphenols, carotenoids, alkaloids, and organosulphur compounds (e.g., glucosinolates) [6].

Polyphenols, which are produced via the shikimic acid pathway in plants, are widely regarded as the most abundant and widespread of plant secondary metabolites, with over 8000 known compounds belonging to this family reported to date [7]. Phenolic compounds exist naturally within plants, fruits and vegetables, cereals, pulses, nuts, and in beverages like red wine, cocoa, tea, and coffee. It is their unique structure, which is composed of one or more phenolic ring and one hydroxyl group, that has been linked to the potential ability of these compounds to mitigate against degenerative diseases in humans [8]. It is this chemical structural composition, which allows phenolic compounds to react with reactive species such as super oxide anions thus protecting against their harmful effects. Phenolic compounds can be broadly grouped into five-sub classes, based on recurring chemical structures shared by compounds belonging to each sub-class, namely phenolic acids, flavonoids, stilbenes, coumarins, and tannins, which is conditional to the quantity of phenolic rings present and the types of elements attached to the aromatic rings [5,8,9].

**Figure 1:**
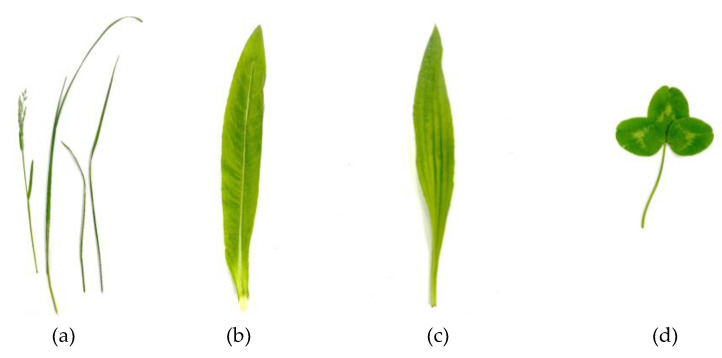
Agricultural grassland crops. (**a**): *Lolium perenne* L, (**b**): *Cichorium intybus* L, (**c**): *Plantago lanceolata* L, (**d**): *Trifolium pratense* L.

There is strong scientific evidence for the contribution of phytochemicals towards health/wellbeing and the mitigation against diseases, however most of the evidence has been gathered from well-recognized sources of these phytochemicals such as fruits and vegetables [3,5,10,11]. As a result of our dependence on animal foods, grassland species cultivated as feedstuffs for meat production represent a considerable proportion of plant biomass present in our biosphere. In recent times, evidence has been accumulating for the potential of grassland species as a source of phytochemicals. Therefore, the focus of this review will highlight the presence of antioxidants in the common grassland crops illustrated in Figure 1, methods that have been used to date to isolate them from these and the beneficial biological effects (antioxidant, anti-cancer, anti-diabetic) the compounds exhibit.

## 2. Grassland as a Source of Polyphenolic Compounds

*Lolium perenne* L., or perennial rye grass, is a member of the *Poaceae* family, which is characterized by green narrow leaves, a blade-like appearance, and a fibrous root system (Figure 1a). *L*. *perenne* is considered high quality and high yielding, and therefore is the most prevalent crop within agricultural farming practices for livestock forage. Despite its abundant use within agriculture, few researchers have measured the phenolic content of rye grass, and for those who have, they primarily focused on the effect of rye grass feeding regimes on the polyphenolic content (terpenoids, phenols, carotenoids, and tocopherols) of meat and dairy products. Besle et al. [12] reported that there was twice as much total phenolics in cow’s milk from cow’s fed with ryegrass pasture versus the phenolic quantity present in the milk of cow’s fed with concentrated feeds. Similarly, Adler et al. [13] demonstrated a three-fold increase in pasture fed versus concentrates, whilst also commenting on the presence of isoflavones, such as daidzein, formononetin, and genistein. Clarke et al. [14] found that raw bovine milk from a cow fed a rye grass diet contained a higher phenol content when compared to the milk of cows which were fed a mixture of grass and clover and total mixed ratio feeds. The authors reported the presence of apigenin, daidzein, genistein, and formononetin in milk from cows on a rye grass diet [14].

*Cichorium intybus* L., or chicory, is a perennial herbaceous plant and a relative to the *Asteraceae* (daisy) family, *Cichorium* genus (Figure 1b). *C. intybus* is cultivated worldwide, predominantly in temperate regions of Europe [15]. *C. intybus* has been incorporated within agriculture for its ability to produce a high quality forage for sheep or dairy production [16]. It is recognized for its nutritional composition which contains proteins, fats, inulin, oils, and other volatile compounds [15,17]. Furthermore, *C. intybus* has demonstrated in vitro anti-inflammatory activity via the inhibition of cytokines [18], antidiabetic and hypoglycemic [19], cardioprotective and nephronprotective effects, as evidenced in animal studies [20]. In addition, *C. intybus* was reported to demonstrate moderate antioxidant behavior and cytotoxic effects on in vitro Caco-2 cells [21].

*Plantago*, or plantain, is a constituent of the *Plantaginaceae* genus, which comprises over 200 known species. The most common varieties grown are *Plantago major* (also known as broad-leaf plantain) and *Plantago lanceolata* (otherwise referred to as narrow leaf plantain, or ribwort). *Plantago* grows throughout temperate climates across Europe and Asia, and is commonly present in grassland, garden lawns, and along roadsides [22]. The plant is considered fast-growing and resilient and is therefore widely used within agriculture grazing systems due to its palatable taste, ability to fortify forage with protein, and reduced incidence of parasitic infection [23]. The plant is identifiable by its tall green, straight leaves with short soft hairs surrounding the leaf (Figure 1c). The edible aerial plant parts have been incorporated into conventional folk medicines for decades, and the plant has since been recognized within British Pharmacopeia as a medically important plant [24]. *Plantago* has been incorporated within holistic medicine to promote the treatment of urinary tract infections, throat, mouth, respiratory, treatment of edema, diuretic and antimicrobial properties [25,26]. These plants have also shown antioxidant and anti-inflammatory effects relating to skin inflammation and wound healing [27], anti-cancerous effects relating to CAL5 breast cancer cells [28,29], and anti-diabetic, and anti-obesity through *P. lanceolata* extract tea [30]. The health-related properties are considered to be modulated by the secondary metabolites within the plant. Some of the polyphenols reported within *Plantago* genus have included chlorogenic acid, quercetin luteolin-7-O-glucoside, rutin, and hesperetin glucoside [30,31].

*Trifolium pratense* L., or red clover, is a member of the legume class which grows straight and elongated. *T. pratense* is identifiable by a 2–3 cm pink-reddish flower head and three oval-shaped green leaves that have a horizontal white division on the upper side (Figure 1d). *T. pratense* has been incorporated into agricultural farming practices for many years due to its strong nitrogen fixation ability and high yielding growth. *T. pratense* has been used for many decades within traditional holistic medicine to treat a variety of illnesses, such as cold and flu symptoms [32]. In addition, anti-inflammatory, antioxidant, anti-cancerous, antibacterial, and estrogenic properties for the treatment of menopausal disorders have been reported with the uptake of *T. pratense* [33,34,35]. Many bioactive compounds (isoflavones) derived from this plant are commercially available as nutritional supplements, primarily in the management of menopause as a natural estrogen replacement [36]. Most researchers are in agreement that the properties associated with *T. pratense* are attributed to their phytochemical composition of phenolic acids, flavonoids, and isoflavones present to include daidzein, genistein, formononetin, and biochanin A [1,14,37].

## 3. Methodology

The search engine Google Scholar was used to identify publications relating to the key words “phenols” and “polyphenols” in “grassland” and “agriculture”. The terms relating to grassland included “perennial rye grass”, “rye grass”, “*Lolium*” “*Lolium perenne*”, “Chicory”, “*Cichorium*” “*Cichorium intybus”*, “Plantain”, “*Plantago”*, *“Plantago lanceolate”*, “Red clover”, “*Trifolium*”, and “*Trifolium pratense*”. In terms of polyphenols, further emphasis was placed on the terms relating to “phenolic acids” and “flavonoids”. In addition, the terminology “antioxidant”, “bioactivity”, “health-benefits”, “anti-diabetic”, and “anti-cancer” were included within the search. Results were filtered by date to identify the most recent publications, however due to the limited number of publications relating to the antioxidant capacity and associated health benefits of grassland crops, only a few old (15–20 years) publications were considered for this review. Many of the papers included within this review originate from the journals published by well-established scientific publishers, including MDPI, Elsevier, Wiley, and American Chemical Society, amongst others. Only papers written in the English language were considered for use within this review.

## 4. Bioactivity

### 4.1. Antioxidant Activity

Natural antioxidants present ubiquitously in plant-based diets and are associated with the mitigation against disease derived from their ability to neutralize reactive oxygen species, such as superoxide and hydroxyl radicals [38,39]. In vitro assays commonly employed to quantify the antioxidant capacity of a sample include the following assays: total phenolic content (TPC), total flavonoid content (TFC), ferric-reducing antioxidant power (FRAP), 2,2′-diphenyl-1-picrylhydrazyl (DPPH•), radical scavenging assay, 2,2′-azino-bis-(3-ethylbenzothiazoline-6-sulfonic acid) (ABTS•), radical cation-based assay, and oxygen radical absorbance capacity (ORAC) assay. A variety of methods have been used to extract antioxidant species prior to their measurement in grassland crops. Supplementary Table S1 (ST1) presents the extraction methods used for *Lolium perenne, Cichorium intybus, Plantago lanceolata*, and *Trifolium pratense* [1,16,20,27,32,33,35,38,40,41,42,43,44,45,46,47,48]. Table 1 outlines the total phenolic and total flavonoid content of commonly cultivated grassland crops and the various chemical assays reported in the literature.

#### 4.1.1. Total Phenolic Content and Total Flavonoid Content

Total phenolic contents (TPCs) are expressed most commonly as gallic acid equivalents and are measured using the well-established Folin–Ciocalteu method. As demonstrated in Table 1, the TPCs from the above-mentioned grassland crops ranged from 1.16 to 152.5 mg gallic acid equivalents (GAE) per gram (g) of dry weight (DW) (Table 1). The highest presence of TPC was reported on *T. pratense* when assessed by Kroyer et al. [33], while the lowest TPC value was reported on *C. intybus* by Kandil et al. [42]. *T. pratense* was reported to have a moderate TPC value of 52.30 mg GAE/g by Kucükboyaci et al. [46].

The total flavonoid content (TFC) assay is often employed to quantify the presence of flavonoid compounds within a given sample. TFCs are typically measured against a reference flavonoid standard measurement, which commonly include quercetin, rutin, or catechin where the results are expressed as milligrams of standard equivalents per gram of dry weight. Table 1 outlines the quantified sum of flavonoids present within the four most common grassland crops in agriculture. The values ranged from 0.167 to 9.6 mg of quercetin equivalents (QE), 5.06 to 26.61 mg of rutin equivalents (RE) and from 10.70–102.42 mg of catechin equivalents (CE). The highest level of TFC was reported by Rapisardra et al. [1], where *P. lanceolata* resulted in 102.42 mg CE/g DW. The lowest TFC was reported by Kandil et al. [42], where *C. intybus* resulted in 0.167 mg of QE/g of DW. However, it should be noted that the use of different flavonoids as reference standards makes the comparison between samples difficult, especially as different species exhibit different quantities of specific flavonoids.

The differences in the TPC and TFC values between species were expected. A number of possibilities may account for the variation within the biomass reservoir such as seasonality. Supplementary Table S1 outlines the harvest location and time of harvest for the common grassland crops focused on in this review. Rapisardra et al. [1] assessed TPC and TFC variability of common grassland crops over a 5-month growing season (April–August) in Ireland. The authors found that each individual crop had optimal growing periods. For instance, *L. perenne* flourished in May 2020 and showed low TPC (29.43 mg GAE/g DW), which may be attributed to the mixture of rain, extended sunlight hours, and increased ground temperatures typical during this time when compared to winter months. On the other hand, *P. lanceolata* and *T. pratense* yielded the highest TPCs in June of that year (138.69 and 47.49 mg GAE/g DW). In terms of TFC, *L. perenne* peaked in April (26.93 mg CE/g DW), while *P. lanceolata* increased in May (102.42 mg CE/g DW) and *T. pratense* in June (21.84 mg CE/g DW). A similar study was performed by Sanna et al. [38], in which the seasonality of *P. lanceolata* was assessed over four time points of the year (October–July), while also assessing the influence of growth location. The authors assessed variation across three site locations: field, quarry 1, and quarry 2. In terms of harvest time, a similar observation was made where the TPC peaked in April for each harvest location (190.9, 230.0, and 240.3 mg GAE/g DW), as did TFC (66.9, 81.2, and 89.6 mg CE/g DW). Field and quarry 2 experienced its lowest TPC values in July (65.2 and 165.1 mg GAE/g DW), while quarry 1 saw the greatest decline in TPC in October (116.6 mg GAE/g DW). TFC adhered to the same trend as TPC in terms of decline (16.3, 37.7, and 43.1 mg CE/g DW). Only values relating to ‘field’ harvest were used within Table 1 to facilitate similarity amongst other field studies. Other studies have commented on their harvest locations (as outlined in Supplementary Table S1)—unfortunately the lack of uniform growing conditions, seasonal influence, environmental factors, and grassland crops harvest make a clear comparison difficult. Soil composition is another such area that affects the growth and development of grassland crops. However, very few publications have investigated this concept sufficiently. To the best of our knowledge, only two publications have commented on the growing conditions of *P. lanceolata* in terms of soil composition. Ahatović et al. [26] observed the ability of *P. lanceolata* to grow in heavy metal soils with high concentrations of nickel, zinc, and chromium. Sanna et al. [38] briefly reported on the presence of carbon, nitrogen, and potassium, as well as pH across the three harvesting sites. The author remarked that of plants harvested within quarry 2, a 25% greater volume of total phenolics, flavonoids and non-flavonoids, tannic and non-tannic were recorded. Nevertheless, further investigations are required to fully evaluate the influence that soils have on grass crops’ phenolic composition.

Following on from the primary growth factors influencing the phenolic composition of grassland crops, the following sources of variation largely presents within the preparation of raw material extraction variation. The extraction table outlined in Supplementary Table S1 states the part of the plant used. The aerial part of the plant, which broadly relates to the section of the plant grown above ground level, to include the stems, petals, flowers, and any seeds or fruits was most commonly used for experimentation throughout grassland crops. Plant roots may also be assessed. The co-author of this review (N.P Brunton) was involved in the phenolic assessment of dandelion (*Taraxcum officinale*) root—a member of the *Asteraceae* family in which *C. intybus* is a relative. The natural root extract demonstrated strong antioxidant potential relating to DPPH• and FRAP assays which could have a role within novel food ingredients, or in the mitigation of disease [39]. The use of different extraction solvents is another major factor in influencing TPC and TFC values. Methanol and ethanol are the predominant solvents used within the extraction of polyphenols from plant extracts. However, many studies have found that using aqueous methanol as the extraction solvent is more efficient at achieving greater TPC and TFC values than using aqueous ethanol. This was apparent in the studies by Kandil et al. [42] and Epure et al. [20], where *C. intybus* extract yielded a higher presence of polyphenols when extracted using methanol instead of ethanol. Few authors have evaluated the use of acid within extractions. Kagan et al. [40] recorded a decrease in TPC values of *L. Perenne* when 10% acetic acid and methanol were used. A similar observation was apparent when 0.5% acetic acid was used with 50% methanol (35 mg GAE/g FW) as opposed to 50% methanol alone (38 mg GAE/g FW) of basil leaves (*Ocimum basilicum* L.) [49].

Another factor responsible for the variations in TPC is the solid to liquid ratio, which varied greatly between the authors, ranging from 1:10 to 1:100 (Supplementary Table S1). In theory, when less volume of solution is available, a reduced surface interaction ability between sample and solvent will exist, thus lowering diffusion of the target compound to the solvent. For example, within the species *P. lanceolata*, Sanna et al. [38] observed a TPC value of 65.2–190.9 mg GAE/g and a TFC value of 16.3–66.9 mg CE/g through a 1:50 weight-to-volume (*w*/*v*) ratio using 80% methanol. Bahadori et al. [44] found a TPC value of 45 mg GAE/g and a TFC value of 9.6 mg QE/g through a 1:12.5 *w*/*v* ratio using pure methanol. Extraction time was another variation factor, as exemplified by the lengthy extraction period performed by Sanna et al. [38], where a 24 h extraction was compared against the 5 h extraction performed by Bahadori et al. [44]. A longer extraction period is considered to facilitate the total exhaustion of polyphenolic acids from the sample, which often results in a greater extraction of polyphenolic yields. However, this does depend on the extraction solvent and technique used.

**Table 1:** Total phenolic content (TPC) and total flavonoid content (TFC) of the four most common grassland crops reported in the literature.

	TPC		TFC				
Grassland Species	mg GAE/g	Method	mg QE/g	mg RE/g	mg CE/g	Method	References
*L. perenne* (Perennial rye grass)	19.95–29.43	100 µL sample/standard. 2 mL 2% Na_2_CO_3_. 100 µL F-C reagent. 30 min incubation in DC. λ 720 nm.			10.70–14.57	250 µL sample/standard. 5% NaNO_3_, 150 µL 10% AlCl_3_, and 0.5 mL NaOH (1M). 30 min incubation. λ 510 nm.	[1]
	26.46	50 µL sample/standard in MeOH mixed with 50 µL F-C reagent, 300 µL of 20% Na_2_CO_3_, and 3.5 mL DiH_2_O. 30 min incubation in DC. λ 728 nm		25.37		2 mL sample/standard in MeOH mixed with 300 µL 5% NaNO_2_, wait 6 min. Add 300 µL of 10% AlCl_3_, wait 6 min, and add 1 of NaOH and add DiH_2_O to make up 6 mL. 15 min incubation. λ 510 nm.	[41]
	12.1	0.16 mL of sample/standard was mixed with a 1:10 dilution of F-C reagent in DiH_2_O with 0.64 mL Na_2_CO_3_ (0.71 M). 30 min incubation in DC. λ 765 nm.					[40]
*C. intybus* (Chicory)	47.50–74.94	See above.			35.56–58.95	See above.	[1]
	23.94	1 mL of F-C was added to 2 mL of both MeOH and EtOH extract solutions, followed by 10 mL DiH_2_O and 25 mL Na_2_CO_3_. 30 min incubation in DC. λ 760 nm.		5.06		10 mL of extract was diluted with 25 mL MeOH. 5 mL of 5% sodium acetate, 8.3% AlCl_3_, and further diluted with MeOH to make up 25 mL. λ 430 nm.	[20]
	15.4	250 µL of sample/standard was mixed with 250 µL of diluted F-C reagent (1:1), 500 µL of 10% Na_2_CO_3_, and 4 mL DiH_2_O. 25 min incubation. λ 740 nm.					[50]
	40.51	F-C reagent was added to the sample, followed by 2% Na_2_CO_3_ in 0.1M NaOH.					[17]
	85	100 g of extract was boiled with 95% EtOH for 2 h and filtered. The residue was dissolved and the remaining extract was subjected to free, acid-and-alkali hydrolysis to quantify polyphenolic compounds.		6.82		1% AlCl_3_ was added to the ethanolic sample extract. λ 510 nm.	[43]
	1.16	200 µL of sample/standard was mixed with 3 mL DiH_2_O, followed by 250 µL F-C reagent and 250 µL of 20% Na_2_CO_3_. 2 h incubation. λ 750 nm.	0.167			0.5 mL of sample/standard was mixed with 0.5 mL of 2% AlCl_3_. 60 min incubation. λ 420 nm.	[42]
*P. lanceolata* (Plantain)	112.27–138.69	See above.			81.01–102.42	See above.	[1]
	65.2–190.9	Determined using F-C reagent method.			16.3–66.9	Quantified using AlCl_3_ method.	[38]
	45	Sample extract (1 mL of 2mg/mL), 45 mL DiH_2_O mixed with 1 mL F-C reagent, 3 mL 2% Na_2_CO_3_. 2 h shake incubation. λ 760 nm.	9.6			1 mL of 2% AlCl_3_ in MeOH. 1 mL of 2 mg sample solution. 10 min incubation. λ 415 nm.	[44]
*T. pratense* (Red clover)	38.60–47.49	See above.			12.62–21.84	See above.	[1]
	46.88	100 µL sample dissolved in MeOH, + 2 mL 2% Na_2_CO_3_. 5 incubation. 100 µL F-C reagent. 30 min incubation in DC. λ 750 nm.		26.61		Sample (50 mg) dissolved in 10 mL 80% MeOH, filtered (125 mm). 300 µL extract, 3.4 mL 30% MeOH, 150 µL 0.5 M NaNO_2_ + 150 μL 0.3M AlCl_3_. 5 min incubation + 1 mL NaOH (1 M). λ 510 nm.	[45]
	152.5	0.5 mL of sample in EtOH was mixed with 0.5 mL of F-C reagent to a flask containing 7 mL DiH_2_O. Wait 3 min. 1 mL of saturated Na_2_CO_3_ was added. DiH_2_O was added to make up to 10 mL in volume. 60 min incubation in DC. λ 750 nm.					[33]
	34.38–48.46	0.5 mL of sample extract was mixed with 1.5 mL of diluted F-C reagent (1:9), and 2 mL of 7% Na_2_CO_3_. 60 min incubation in DC. λ 765 nm.		20.98– 21.18		0.1 mL sample/standard was added to 1 mL of 96% EtOH, 0.05 mL of 33% acetic acid, 0.15 mL 10% AlCl_3_ and 2.0 mL 5% hexaethylenetetraamine solutions. 30 min incubation. λ 475 nm.	[35]

TPC: total phenolic content; TFC: total flavonoid content; mg GAE/g: milligram gallic acid equivalent per gram; MeOH: methanol; EtOH: ethanol; QE: quercetin equivalent; RE: rutin equivalent; CE: catechin equivalent; F-C: Folin-Ciocalteau reagent; Na_2_CO_3_: sodium carbonate; NaNO_3_: sodium nitrate; AlCl_3_; aluminium chloride; DiH_2_O: deionised water; DC: darkened conditions; Weights are displayed as dry weights of extract.

Table 2 displays the level of individual (a) phenolic acids and (b) flavonoids which have been reported within the common grassland crops. In relation to *L. perenne*, very few compounds were identified within literature. This may be due to the lack of interest in the crop beyond its uses within agricultural feeding practices. As far as we know, only one author has measured the phenolic acid and flavonoid composition of *L. perenne* [1]. Chlorogenic acid was the major phenolic acid identified within this crop. Individual flavonoids within this crop presented at low quantities, including biochanin A, daidzein, kaempferol, luteolin, naringenin, and genistein. The low presence of compounds identified within this study contributed to the low TPC and TFC values presented within Table 1. However, this is most likely due to the type of analysis performed by the authors. Targeted analysis was performed using LC-ESI-MS/MS, which was used to identify eight compounds. The co-authors of this review (D.K Rai and N.P Brunton) performed similar research in characterizing the phenolic composition *of Lamiaceae* spp. through using LC-ESI-MS/MS analysis. The authors identified 38 phenolic compounds within five *Lamiaceae* species following their extraction in 80% methanol (1:25 (*w*/*v*)) [51]. This type of untargeted analysis facilitated the characterization of a greater variety of polyphenols, to include hydroxycinnamic acid and hydroxybenzoic acid derivatives, phenols, and flavonoids, which is paramount in identifying potentially beneficial novel compounds [51]. In addition, N.P Brunton was involved with the identification of 18 phenolic compounds from dandelion (*Taraxcum officinale*) root, from of a database of 36 compounds, to include chlorogenic acid, syringic acid, caffeic acid, apigenin, luteolin, and naringenin derivatives. [39]. As the name suggests, *C. intybus* is a rich source of the phenolic acid—chicoric acid (1692–18,450 µg/g), as well as chlorogenic acid (910–1208 µg/g) and mono-caffeoyltartaric acid (742–1323 µg/g). Kaempferol glucuronide, luteolin, and biochanin A (876.2, 874, 866 µg/g) are some of the most dominant flavonoids presented within the plant. *P. lanceolata* has been well reported on the presence of phenolic compounds such as verbascoside (45,139–95,000 µg/g), chlorogenic acid (7115–7588 µg/g), and caffeic acid (90.46–1860 µg/g). Reported flavanols presented within this crop include formononetin, biochanin A, and luteolin (440, 166,118 µg/g, respectively). *T. pratense* is a well-established in the management of menopause through supplementation. This is due to its unique composition of polyphenols, such as the phenolic acids—chlorogenic acid and ferulic acid (78–17 µg/g), with a rich source of flavonoids and isoflavonoids—formononetin, biochanin A, and daidzein (5934, 2372, and 262 µg/g, respectively). Collectively, the above-mentioned phenolic acids and flavonoids have contributed to the overall total phenolic and total flavonoid content of the species, as demonstrated in Table 1.

### 4.2. Bioactivities of Grassland Crops

Many studies have demonstrated the influence that plant derived phytochemicals have on the human health through enhanced levels of antioxidants within the bloodstream and in the mitigation and protection against oxidative stress [41]. Fruits and vegetables are excellent sources of natural origin phytochemicals such as polyphenols (example: catechins, naringenin, quercetin, resveratrol and chlorogenic acids) [8], but are not available at the biomass levels that plant sources, such as those of grassland origin are. Therefore some researchers have investigated the potential chemo-protective and glucose-lowering action of grassland species as natural, cost-effective and accessible means of mitigating the risk of diseases such as cancer and diabetes, respectively [53,54,55,56,57].

#### 4.2.1. Antioxidant Activity

A limited body of evidence exists relating to the phytochemical and antioxidant properties of grassland species, which are outlined in Table 3. The FRAP assay is commonly employed to determine the antioxidant power of the sample extract. The FRAP values in four grasses ranged from 67.36 to 896.68 µM Trolox equivalent (TE)/g. The highest FRAP activity was reported in Romanian *C. intybus* at 896.68 µM TE/g [20], while the lowest value was recorded in *L. perenne* species harvested in August 2020, Ireland [1]. The DPPH• radical scavenging assay may be displayed by a series of unit measurements. For example, DPPH• activity in *C. intybus* exhibited strong activity when performed by Sahan et al. [17] using 15 mL MeOH containing 4% formic acid. However, the DPPH• assay is more commonly expressed as percentage inhibition. A high percentage inhibition was shown by *P. lanceolata* (80.94%, 0.25mg/mL), while the lowest value was shown by *L. perenne* (19.27%, 0.25 mg/mL) [1]. *C. intybus*, on the other hand, displayed a moderate DPPH• activity (54.65%, 1 mg/mL) when extracted in ethanol [42], despite its highest FRAP activity being reported by Epure et al. [20]. Possible reasons for such differences may relate to the extraction concentration used, in addition to the attributes mentioned above in Section 4.1.1. Some authors have reported DPPH• activity as EC50 values, which relates to the Effective Concentration (EC) of samples required to obtain 50% DPPH• inhibition at different time-points. In contrast to the above values, a low EC50 value denotes a high antioxidant capacity. Of the literature sampled, only values for *T. pratense* were obtained, in which the highest antioxidant capacity values were reported by Esmaeili et al. [45] through in vivo (94.25 µg/mL) and in vitro (205.47 µg/mL) experimentation. Kroyer et al. [33] found the least potent effect when evaluating red clover extract in aqueous–alcoholic solution (320 µg/mL). The IC50 values relate to a sample or analyte concentration required to inhibit DPPH• activity by 50%. Similarly to that of the EC50 value, low IC50 indicates a strong potency at lower concentrations. *L. perenne* achieved a strongest potency of the literature sampled at 0.417 mg/mL [41], which is in contrast to the inferior antioxidant activity of this species mentioned previously. IC50 values were followed by *P. lanceolata* at 4.2 mg/mL [27], while the least effect was noted through using *C. intybus* at 67.27 mg/mL [43].

The ABTS• assay is similar to the DPPH• assay, where a radical cation decolourization ability is used to establish the antioxidant capacity. In terms of ABTS• values within grassland, Iqbal et al. [48], reported a value of 0.43 mg ascorbic acid equivalents (AAE)/g of fresh weight (FW) in *L. perenne.* Sanna et al. [38] assessed the seasonal influence of field-grown *P. lanceolata* over a 4-month timeframe to yield an average value of 146.3µmol TE/g DW. Sahan et al. [17] investigated *C. intybus* to establish a value of 161.80µmol TE/g DW. Furthermore, Esmaeili et al. [45] evaluated the ABTS• capacity of *T. pratense* in methanol through in vivo and in vitro assays that produced values of 111.84 and 351.46 µg/mL, respectively. A lack of a uniform unit measurement persists within the published data relating to grassland samples. Therefore, a standardized unit measurement is recommended throughout antioxidant assays.

Another such well-regarded antioxidant assay, especially in food science, is the ORAC assay, which quantifies how effectively a compound scavenges reactive oxygen species present by donating hydrogen ions. As oxygen is an abundant compound within the human body, the ORAC assay has been considered the closest to human physiology compared to any other assay, which may be of benefit when evaluating potential bioactive and health properties associated with grassland species, such as anti-inflammatory, anti-cancerous, or anti-diabetic properties. To our knowledge, Rapisarda et al. [1] were the only authors to report ORAC values relating to the desired four common grasses. The author published data ranging from 915.26 to 2267.28 µM TE/g, with *L. perenne* producing the lowest and *P. lanceolata* achieving the highest ORAC values. *C. intybus* achieved a moderate result of 1343.266 µM TE/g [1].

Evident variation within antioxidant activity persists between the grassland species. A number of reasons may contribute towards such variation, such as the variety of cultivars used and contrasting growth locations, much of which is mentioned in the above section and outlined in *ST1*. Additionally, plant origin, the part of the plant used (aerial parts or root), and environmental growth conditions will influence the rate pasture plants will grow. This will influence the presence of plant secondary metabolites (including polyphenols) and their bioactivity [7]. As individual grassland crops have specific requirements, a comparison between grassland crops as a whole is not just possible to compare multiple results from different studies, due to the huge degree of variation exists between the factors outlined. For instance, spring pastures, such as *Lolium* rye grass crops, tend to grow at a greater rate and produce a higher volume of dry matter when conditions are favorable, such as within temperate conditions with sufficient rainfall. Crops like *Cichorium* and *Plantago* spp. thrive during the summer period to achieve similar dry matter.

A greater understanding of the phytochemical composition, such as the presence of phenolic acids or flavonoids, may provide further insight into antioxidant activity, as discussed in the above section. The evaluation of optimal conditions is one such way to evaluate this. The use of response surface methodologies is encouraged. This consideration is optimal for nutrient composition and for the secondary metabolites of plants. The identification and selection of optimal growth parameters and extraction conditions is important as it will influence multiple parameters, such as influencing reducing power and its ability to donate electrons or hydrogens. Furthermore, individual phenolic compounds may have different influences on the antioxidant activity of plants. Bahadori et al. [44] commented on the association of phenolic compounds, strong scavenging properties, and high reducing power of *P. lanceolata*. The plant was identified as a powerful natural agent which has the ability to reduce oxidative stress through chelating capacity and electron and hydrogen donation mechanisms [44]. Ultimately, the most reliable way to evaluate polyphenolic composition is by using analytical methods such as liquid chromatography or mass spectrometry analysis. Several authors cited in Table 2 have used analytical techniques such as liquid chromatography and mass spectrometry to successfully identify and quantify the individual polyphenols in the grassland crops.

#### 4.2.2. Anti-Cancer

Cancer represents one of the most common causes of mortality globally. Many plants have been evaluated as a means of establishing a cure for the disease. *P. lanceolata* is well established within traditional folk medicine, which provoked further investigation into the potential cytotoxicity of the plant. Alsaraf et al. [28] evaluated the efficacy of a *P. lanceolata* concentrate against breast cancer cells. The extract selectively inhibited the progression of CAL51 triple-negative breast cancer cells, and highlighted minor effects on MCF7, AMJ13, or MDAMB cells. In addition, the author assessed the presence of phenolic bioactive compounds to identify rutin (12.27–13.37 µg/mL), myricetin (11.88–15.27 µg/mL), quercetin (10.23–16.99 µg/mL), and kaempferol (11.42–12.16 µg/mL) within the plant extract, which may contribute towards the inhibitory effects relating to breast cancer cell proliferation [28]. A similar outcome was achieved by Abate et al. [29] in which *P. lanceolata* L. decreased proliferation of CAL51 triple-negative breast cancer cells with a minor influence on MCF7, AMJ12, and MDAMB breast cancer cells. Azzini et al. [21], assessed the relationship between *Cichorium intybus* L. (Variegated Chicory of Castelfranco) extract in in vitro Caco-3 cell models and demonstrated moderate antioxidant activity up to concentrations of 17µM. In addition, the flavonoid content was assessed using HPLC-ECD, which revealed the presence of quercetin (14.20 ± 4.51 mg/kg), kaempferol (11.80 ± 3.64 mg/kg), and apigenin (3.59 ± 0.29 mg/kg) [21]. Eray et al., [53] concluded that *C. intybus* L. extract from both the shoot (0.64 mg/mL) and leaf (0.69 mg/mL) had a high cytotoxic effect in HepG2 hepatocellular carcinoma cell lines. Akbaribazm et al. [54], assessed the effects of *T. pratense* on 4T1 breast tumor-bearing BALC/c mice. Over the 36-day period, supplementation inhibited the growth of 4T1 tumor cells at a dosage of up to 400 mg/kg. A significant decrease in serum level E2—a known stimulus for breast cancer proliferation—and an increase in serum level IL-12 and IFN- γ were reported [54]. Furthermore, *T. pratense* exhibited cytotoxic effects against ovarian cells derived from Chinese hamsters (CHO-K1) at concentrations of 100 μg/mL when extracted under microwave-assisted conditions [55]. Collectively, an array of beneficial outcomes exist that relate to anti-cancerous properties associated within plants of grassland origin.

#### 4.2.3. Anti-Diabetic

Centers for Disease Control and Prevention have stated that 34.2 million people worldwide are effected by Type 2 diabetes (T2DM), which is typically controlled through the oral administration of pharmaceutical drugs, dietary, and lifestyle modifications [58]. The inclusion of plant-derived phytochemicals can be valuable in the treatment of diabetes. Polyphenols are considered to mimic pharmacological drug action through the inhibition of radical species to create compounds that retard reactions between other molecules to increase insulin secretion, preventing hyperglycemia [30].

Polyphenol-rich grassland extracts have demonstrated beneficial outcomes on the management of diabetes in laboratory animals. Dalar and Konczak [30] evaluated the inhibitory activities of a *P. lanceolata* herbal tea infusion and identified a strong potential as a natural anti-diabetic, anti-obesogenic and cardiovascular agent. The tea demonstrated the in vitro suppression of the enzymes α-glucosidase, pancreatic lipase (IC50 1.43 ± 0.04, and 4.53 ± 0.08 mg/mL, respectively), and α-amylase (32.35 ± 0.43% inhibition), all of which are strongly associated within metabolic syndrome and diabetes mellitus [30]. In a follow-on study performed by Dalar and Konczak [59], herbal tea containing *C. intybus* showed inhibitory action towards α-glucosidase (IC50 4.25 ± 0.08 mg/mL), and suppressed pancreatic lipase (IC50 3.97 ± 0.20 mg/mL), which may demonstrate anti-diabetic potential. The authors suggested that such in vitro activity may be attributed to the presence of chlorogenic acid (4-O-caffeoylquinic acid) (10.9 ± 0.2mg/g DW), caftaric acid (6.2 ± 0.1mg/g DW) and chicoric acid (16.2 ± 0.3 mg/g DW), and luteolin hexoside (7.8 ± 0.2 mg/g DW) present within that sample [59]. Chlorogenic acid, in particular, has been recognized as a potential aid in improving glucose regulation of T2DM patients, however research is ongoing [60,61].

Pushparaj et al. [19] evaluated the hypoglycemic effect of *C. intybus* on streptozotocin (STZ)-induced diabetic rat models in which 125 mg/kg BW of *C. intybus* was fed for 14 days. A reduced hepatic glucose-6-phosphate activity was observed within the treatment versus control group, however no change in serum insulin was recorded [19]. Thi Viet Huong et al. [56] investigated the potential for *P. major* for the treatment of STZ-induced, diabetic mice models. The animals received 400 mg/kg BW of plant extract, followed by the oral administration of 1.25 g/kg BW of glucose. A 27% reduction in blood glucose was recorded after 120 min versus the control, whilst within the non-diabetic cohort a 23% reduction was recorded. Additionally, the rate of reduction was comparable to the oral supplementation of 70 mg/kg BW of the anti-hyperglycemic drug ‘Glucophage’ following 15 days of treatment. In the non-diabetic cohort, the reduction was comparable to that induced by the oral administration of glibenclamide—a pharmacological drug belonging to a class known as sulfonylureas, primarily used to lower blood sugar in T2DM patients [56]. The influence of isoflavones derived from *T. pratense* was studied by Parandin et al. [57] to evaluate antidiabetic and hepatoprotective effects. The results demonstrated that in diabetic mice treated with 500 and 750 mg/kg BW of *T. pratense* for 15 days, a significant (*p* < 0.0001) reduction in serum glucose and a decline in liver enzyme aspartate aminotransferase levels was observed, concluding a significant decrease versus the control cohort [57].

It is apparent that dietary polyphenols could have a role in the management of T2DM. However, to the best of our knowledge, evidence for anti-diabetic potential relating to polyphenols of grassland origin remains limited to in vitro and animal studies. A number of randomized clinical human trials have provided evidence for the efficiency of the inclusion of polyphenol rich foods, such as wine [62], cocoa [63], and oat ingestion [64] into diets, to impart anti-diabetic effects such as blood pressure, glucose metabolism, and vascular function. The favorable improvements are likely due to improved insulin secretion and sensitivity. Many of the polyphenols present within such samples mirror that of the polyphenolic compounds within grassland species and a possible comparison could be drawn.

## 5. Conclusions

Agricultural grassland crops contain a rich variety of phytochemicals, particularly polyphenols, which have the potential to exert beneficial biological properties, where the inclusion of such plant-derived compounds within the diet may contribute towards overall human health. This present review outlines the phenolic composition, bioactivity (antioxidant anti-cancer, and anti-diabetic activity) relating to the aerial parts of *Lolium perenne* L., *Cichorium intybus* L., *Plantago lanceolata* L., and *Trifolium pratense* L. Such crops have demonstrated efficacy within both in vivo and in vitro assays, and certain trials relating to antioxidant, anti-cancer, and anti-diabetic outcomes.

However, it should be noted that the outcome relies heavily on the extraction protocol implemented. For instance, solvent:liquid ratio, time, and temperature are the most common factors to influence TPC yield, thus it is recommended to optimize such parameters depending on the sample. In addition, the use of acetic acid is inadvisable as it has reported a negative effect on TPC yields.

Literature has demonstrated a considerable potential for plant polyphenols with specific reference to the composition and associated bioactivities of agricultural grassland. Grassland crops have a promising future within value-added food and nutraceutical innovation products. Furthermore, the use of plant-derived medicinal components is ongoing within cancer prevention and treatment, and in the management of T2DM. Grassland crops are considered non-toxic, with fewer reported side effects, and are therefore well tolerated by patients, making the inclusion of the above-mentioned grassland crops a potentially suitable anti-cancerous and anti-diabetic agent. The findings outlined within this review paper give new guidance for future investigations into the therapeutic uses of grassland crops to fully appreciate their potential. Despite the collection of literature included within this study, a lack of trials exists relating to the safety of such a product. Therefore, it is recommended that further works would include toxicity investigations in order to understand its role and contribution to human health benefits. Overall, grassland crops present a valuable, yet underutilized, source of polyphenols that could be of huge benefit to human health.

## Figures and Tables

**Table 2:** Reported concentrations expressed in µg/g DW of (**a**) phenolic acids and (**b**) flavonoids within grassland species. Different colours represent the concentrations of each compound in different grassland species.

(a) Phenolic Acids	*L. perenne*	*C. intybus*	*P. lanceolata*	*T. pratense*	Reference
3-Hydroxybenzoic acid			4		[44]
4-Hydroxybenzoic acid			36		[44]
			149.46		[27]
				0.713	[47]
2,5-Dihydroxybenzoic acid			16.2		[27]
				0.088	[47]
Caffeic acid		20			[17]
			90.46		[27]
			1860		[50]
				1.041	[47]
Chicoric acid		2334			[52]
		18450			[20]
		1692			[16]
Chlorogenic acid	4668	1208	7588	78	[1]
		910.69			[20]
			7115.62		[27]
Cinnamic acid		115			[16]
			209.78		[27]
Ferulic acid		10			[17]
			57.01		[27]
				17	[44]
Gallic acid		38.17			[16]
		0.03			[17]
			212.01		[28]
Mono caffeoyltartaric acid		1323			[52]
		742.81			[20]
p-Coumaric acid		0.03			[17]
		24			[44]
			87.67		[27]
Protocatechuic acid			103.48		[27]
				0.197	[47]
Vanillic acid		0.08			[17]
			411.52		[27]
Verbascoside			95,000		[44]
			45,130		[38]
Syringic acid		0.23			[17]
**(b) Flavonoid class**	***L. perenne***	***C. intybus***	***P. lanceolata***	***T. pratense***	**Reference**
Apigenin			184.38		[27]
			27		[44]
				0.32	[47]
Apigenin 7-*O*-glucoside			21		[44]
				0.288	[47]
Apiin			3.48		[27]
Biochanin A	20	866	166	2372	[1]
Catechin		20			[17]
			23		[44]
Daidzein	26	154	12	262	[1]
				1.527	[47]
Epicatechin gallate		29.28			[16]
Formononetin	38	2646	440	5934	[1]
Genistein		641.8			[16]
				0.588	[47]
Hesperidin		0.17			[17]
			270		[44]
Hyperoside			2.65		[27]
			290		[44]
				45.259	[47]
Isoquercitrin		427.3			[20]
Kaempferol	22	852	74	36	[1]
				0.062	[47]
Kaempferol-3-*O*-glucoside				8.544	[47]
Kaempferol glucuronide		876.2			[52]
Kaempferol malonyl glucoside		341.8			[52]
Kaempferol-3-*O*-gluconide			5.95		[27]
Luteolin	18	874	118	44	[1]
Luteolin-3-glucoside		1390			[50]
Methyl quercetin glucuronide		255			[52]
Myricetin		0.05			[17]
Naringenin	16	14	10	12	[1]
				0.062	[47]
Quercetin	14	16	8	8	([1]
				0.128	[47]
Quercetin-3-*O*-glucoside				24.243	[47]
Quercetin malonyl glucoside		695.3			[52]
Rutin			4.7		[44]
				0.157	[52]
The concentration values are depicted by colour, where green indicates a high concentration per compound, while red indicates a low concentration.					
					
Low	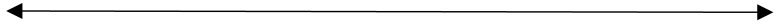				High

**Table 3:** Antioxidant capacity of grassland crops reported in the literature.

Grassland Species	FRAP (µM TE/g)	DPPH•				ABTS• (µmol TE/g DW)	ORAC (µmol TE/g DW)	References
		Activity (µmol TE/g DW)	IC 50 (μg/mL)	% Inhibition	Sample Concentration (mg/mL)			
*L. perenne* (Perennial rye grass)	67.36–102.77			19.27–25.87%.	0.25		755.08–1053.55	[1]
			417.25					[41]
*C. intybus* (Chicory)	194.44–286.71			35.74–58.98%	0.25		1224.33–1594.22	[1]
	896.68		164.98					[20]
		233.66				161.8		[17]
			67.27					[43]
				EtOH: 70.83%. MeOH: 54.65%.	1			[42]
*P. lanceolata* (Plantain)	368.70–482.49			71.59–80.94%	0.25		2032.51–2478.93	[1]
	102–351					87–214		[38]
			4.2					[27]
				51–95%	0.20–0.80			[44]
*T. pratense* (Red clover)	89.54–136.05			23.36–37.58%	0.25		988.17–1220.49	[1]
			94.25					[45]
			320					[33]

FRAP: ferric-reducing antioxidant power; DPPH•: 2,2′-diphenyl-1-picrylhydrazyl; ABTS•: 2,2′-azino-bis-(3-ethylbenzothiazoline-6-sulfonic acid); ORAC: oxygen radical absorbance capacity; TE: Trolox equivalents; DW: dry weight; EtOH: ethanol; MeOH: methanol.

## Supplementary Materials

The following are available online at: https://www.mdpi.com/article/10.3390/foods12224122/s1, Table S1. Extraction methods used for polyphenols from grassland species as reported within literature.
