# Supplementary material for: Polyphenols in Agricultural Grassland Crops and Their Health-Promoting Activities—A Review
Source: Foods. 2023 Nov 14;12(22):4122. doi: 10.3390/foods12224122 (PMC10670144; doi:10.3390/foods12224122)
Supplement: Supplementary file 1 [file foods-12-04122-s001.zip › foods-2672611-supplementary.pdf]

foods

Review

Polyphenols in Agricultural Grassland Crops and Their Health-Promoting Activities—A Review

Emily P. Verhulst [1,2], Nigel P. Brunton [2] and Dilip K. Rai [1,*]

[1] Department of Food BioSciences, Teagasc Food Research Centre Ashtown, D15 KN3K Dublin, Ireland; emily.verhulst@teagasc.ie
[2] The School of Agriculture and Food Science, University College Dublin, Belfield, Dublin D04V1W8, Ireland; nigel.brunton@ucd.ie
* Correspondence email: dilip.rai@teagasc.ie; Tel.: +353-1-805-9569

Table S1. Extraction methods used for polyphenols from grassland species as reported within literature.

Grassland Species	Harvest location and period	Part of plant	Solvent (%)	w/v ratio	Extraction conditions		References
					Temp °C	Time	
L. perenne (Perennial rye grass)	Kentucky, USA. April & May 2014.	Aerial	85% MeOH + 10% acetic acid	1:70			[40]
	Sohag, Egypt. March 2020.	Aerial	80% MeOH				[41]
C intybus (Chicory)	Romania. August 2018.	Aerial	70% MeOH	01:10	60 °C	0.5 hrs	[20]
	Hamilton, Australia. 2018.	Aerial	80% EtOH		4 °C	0.33 hrs	[16]
	Bertinoro, Italy.		60% EtOH	1:15	40 °C	0.25 hrs	[32]
	Cairo, Egypt. March 2017.	Aerial	80% MeOH, & 80% EtOH.		10 °C	25 hrs	[42]
		Aerial	100% MeOH	1:10	RT	Overnight	[43]
P lanceolata (Plantain)	Sardinia, Italy. October 2020, January, April & July 2021.	Aerial	80% MeOH	1:50		24 hrs	[38]
	Mountain of Fruska Gora, Serbia. June 2009.	Aerial	40% MeOH	1:10	RT	72 hr	[27]
	Isparta Provence, Turkey. May 2017.	Aerial	100% MeOH	1:12.5		5 hrs	[44]

T pratense ((Red clover)	Grown *In Vivo* and *In Vitro*. Seeds purchased from Mudrock, USA.	Aerial	95% MeOH	01:40	RT	48 hrs	[45]
	Australia.	Aerial	EtOH	1:7.5		4 hrs	[33]
	Kupiškis district, Lithuania. September 2020.	Aerial	50% EtOH	1:33	100 °C	1 hr	[35]
	Tufanbeyli, Turkey. 2006	Aerial	80% MeOH	1:100	85 °C	0.25 hrs	[46]
	Vojvodina Province, Serbia.	Aerial	70% MeOH	1:10	70 °C	0.33 hrs	[47]
L. perenne and *P lanceolata.*	Spring, Victoria, Australia.	Aerial	80% EtOH	1:2.5	4 °C	12 hrs.	[48]
L. perenne, C. intybus, P. lanceolata and *T pratense*.	April-August 2020. Kildare, Ireland.	Aerial	50% MeOH	1:75	40 °C.	2 hrs	[1]

MeOH; Methanol, EtOH; Ethanol, RT; room temperature, min; minute, hrs; hours.
